# Detection of infectious bronchitis virus serotypes by reverse transcription polymerase chain reaction in broiler chickens

**DOI:** 10.1186/2193-1801-2-36

**Published:** 2013-01-31

**Authors:** Mohammad Jahantigh, Saeed Salari, Mahdi Hedayati

**Affiliations:** Department of Poultry Diseases, School of Veterinary Medicine, University of Zabol, P.O. Box 98615–538, Zabol, 9861335856 Iran; Department of Pathobiolgy, School of Veterinary Medicine, University of Zabol, P.O. Box 98615–538, Zabol, 9861335856 Iran; Department of Clinical Sciences, School of Veterinary Medicine, University of Tehran, Tehran, Iran

**Keywords:** Broiler chickens, Infectious bronchitis virus, Serotypes, RT-PCR

## Abstract

Infectious bronchitis (IB) is a highly contagious disease of the respiratory and urogenital tract of chickens, caused by infectious bronchitis virus (IBV), a member of the family *Coronaviridae*. The disease is common throughout the world where chickens are produced commercially. PCR on reverse transcribed RNA is a potent technique for the detection of IBV. In comparison with classical detection methods, PCR-based techniques are both sensitive and fast. Dozens of serotypes and genotypes of IBV have been detected, and many more will surely be reported in future. This research was conducted to identify the infectious bronchitis virus with group specific primers of avian Coronaviruses in Zabol, southeast of Iran. Tracheal swabs were collected from eleven commercial broiler flocks and these swabs were used for RNA extraction. General primers included XCE2+ and XCE2- that amplify all IBV serotypes were used. Primers MCE1+, BCE1+ and DCE1+ was used to amplifying the specific nucleotide sequence of Massachusetts, 4/91 and D274 serotypes, respectively. The results of this study showed that 36.36% of the sampled flocks were positive to IBV by RT-PCR. Moreover, the Massachusetts was the identified serotype of infectious bronchitis virus. The results provide the first molecular evidence for the presence of infectious bronchitis virus and Massachusetts serotype in Zabol.

**Electronic supplementary material:**

The online version of this article (doi:10.1186/2193-1801-2-36) contains supplementary material, which is available to authorized users.

## Introduction

Infectious bronchitis (IB), also called avian infectious bronchitis, is a common, highly contagious, acute, and economically important viral disease of chickens caused by *coronavirus* infectious bronchitis virus (IBV). The virus is acquired following inhalation or direct contact with contaminated poultry, litter, equipment or other fomites. Vertical transmission of the virus within the embryo has never been reported, but virus may be present on the shell surface of hatching eggs via shedding from the oviduct or alimentary tract. Dozens of serotypes and genotypes of IBV have been detected, and many more will surely be reported in future. The highly transmissible nature of IB and the occurrence and emergence of multiple serotype of the virus have complicated control by vaccination (Saif et al. 2008). To monitor the existing different IBV serotypes in a geographical region, several tests including virus isolation, virus neutralization, hemagglutination inhibition, ELISA and RT-PCR have been employed (Haqshenas et al. 2005; Saif et al. 2008). The ELISA assay is a convenient method for monitoring of both the immune status and virus infection in chicken flocks. Several commercial ELISA kits for IBV specific antibodies detection are already available, which used inactivated virions as coating antigen (Zhang et al. 2005). PCR on reverse transcribed RNA is a potent technique for the detection of IBV. In comparison with classical detection methods, PCR-based techniques are both sensitive and fast (Zwaagstra et al. 1992). Samples for IBV isolation must be obtained as soon as clinical disease signs are evident. Tracheal swabs are preferred and are placed directly into cold media with antibiotics to suppress bacterial and fungal growth and preserve the viability of the virus (Swayne et al. 1998). In Iran, IB is one of the most important viral respiratory diseases of broiler chickens. However, only the Massachusetts vaccine strain is officially authorized. Despite the use of the IBV vaccine it is common to find IBV problems in vaccinated chickens, causing a tremendous economic impact (Nouri et al. 2003). Several serotypes of infectious bronchitis virus have been reported from different parts of Iran (Seyfi-Abad Shapouri et al. 2002; Nouri et al. 2003; Shoushtari et al. 2008). There is no report about the serotypes and molecular detection of IBV in Zabol in the southeast of Iran. The aim of this study was molecular detection of IBV and the IBV serotypes in Zabol.

## Materials and methods

A total of eleven commercial broiler flocks on age 5–7 weeks in Zabol in the southeast of Iran were selected randomly for tracheal swab samples preparation. These flocks were vaccinated against IBV with a live attenuated vaccine during the first week of life. Five swabs were prepared from each flock and each swab was placed in a sterile tube containing PBS (Phosphate Buffer Saline) and was transferred to the laboratory in cold conditions. The swabs from each flock were placed in 1 ml PBS (pH 7.2) and were scraped on the side of the tube to facilitate removal of contents from the swab head. For extraction and purification of viral RNA, 200 μl of prepared sample was used and the procedure of viral RNA extraction and purification kit was utilized (High Pure Viral RNA Kit®, Roche, Germany). Briefly, 200 μl of the sample was added to a 1.5 ml sterile RNase and DNase free microtube containing 400 μl of working solution (polyA plus binding buffer) and processed as recommended by manufacturer. The extracted viral RNA was contained in a 1.5 ml sterile RNase and DNase free microtube and stored at −70°C until further use.

RT-PCR: a commercial cDNA synthesis kit (2-steps RT-PCR kit, RTPL12®, vivantis, Malaysia) was used. The recommended procedures of manufacturer with some modifications were utilized. Briefly, 8 μl RNA, 1 μl of Random Hexamer primer and 1 μl dNTPs were added to a 0.2 ml microcentrifuge tube, boiled for 4 min and cooled on ice for 2 min. 2 μl of 10x RT buffer, 1 μl M-MULV reverse transcriptase enzyme (200 u/μl), and 7 μl nuclease free water were added. The tube incubated 10 min at 25°C, 60 min at 42°C and 5 min at 85°C. Finally cooled on ice and stored at −20°C.

**Figure 1:**
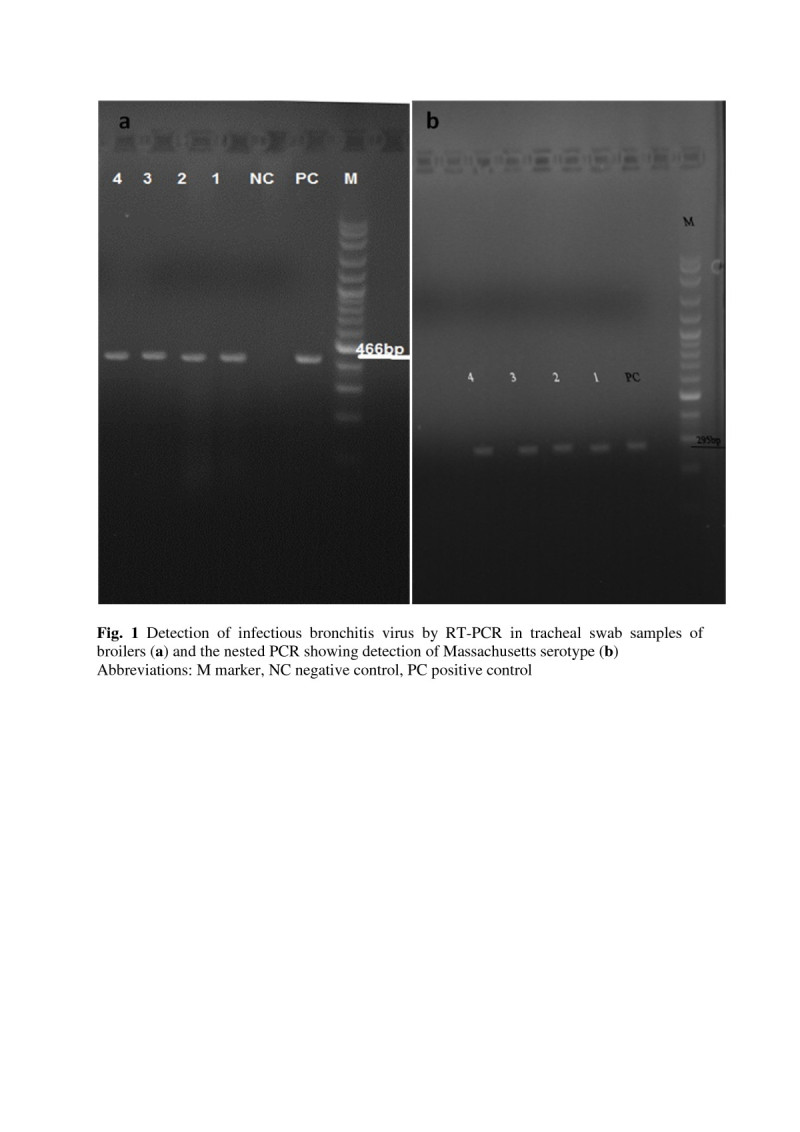
**Detection of infectious bronchitis virus by RT-PCR in tracheal swab samples of broilers (a) and the nested PCR showing detection of Massachusetts serotype (b).** Abbreviations: M marker, NC negative control, PC positive control.

Synthesized cDNA was amplified by PCR reaction. PCR was performed in a reaction volume of 25 μl containing: 2.5 μl PCR reaction buffer, 0.5 μl dNTPs, 0.75 μl of each of XCE2+ and XCE2- primers, 0.75 μl MgCl2, 0.2 μl *Taq* DNA polymerase, 3 μl cDNA and 17.25 μl H2O. Amplification was carried out in a thermocycler using 35 cycles consisting of denaturation for 30 s at 94°C, annealing for 1 min at 55°C, and extension for 1 min at 72°C, followed by a final extension for 7 min at 72°C. Electrophoresis of amplified products was carried out using 1.5% agarose gel. The amplified cDNA fragment were visualized and photographed under UV light. A pattern with 100 bp ladder was used (Figure 1a).

Nested PCR: For serotype identification, a type-specific nested PCR was conducted. Oligonucleotide primers included MCE1+, DCE1+ and BCE1+ respectively specific for a hypervariable region in the *S1* gens of serotypes Massachusetts, D274 and 4/91, and primer XCE3- common for the three serotypes were used (Adzhar et al. 1997; Nouri et al. 2003; Roussan et al. 2008). Table 1 show the sequences of primers which used in PCR reactions. Nested PCR reaction contained 1μl of RT-PCR product of positive reaction, 5 μl PCR buffer, 1μl dNTPs, 1.5 μl MgCl2, 0.25 μl *Taq* DNA polymerase, 34.5 μl H2O and 1 μl of each of the oligonucleotides primers XCE3-, MCE1+, DCE1+ and BCE1+. The thermalcycler parameters were the same as mentioned above. To fractionate DNA fragments, 10 μl of PCR product was loaded onto 1.5% agarose gel and electrophoresed for 55 min in TAE buffer. DNA marker of 100 bp was as size marker for determination of the length of the amplified fragments (Figure 1b).

**Table 1:** **Sequence of oligonucleotides used as primers in the RT-PCR and PCR to identify infectious bronchitis virus (IBV) and IBV serotypes**

Primer	Sequence (5^′^-3^′^)	Amplication fragment (bp)	References
XCE2+	5^′^-CACTGGTAATTTTTCAGATGG-3^′^	466	Adzhar et al. 1997
XCE2-	5^′^-CCTCTATAAACACCCTTGCA-3^′^	466	Adzhar et al. 1997
XCE3-	5^′^-CAGATTGCTTACAACCACC-3^′^		Adzhar et al. 1997
DCE1+	5^′^-TTCCAATTATATCAAACCAGC-3^′^	217	Adzhar et al. 1997
MCE1+	5^′^-AATACTACTTTTACGTTACAC-3^′^	295	Adzhar et al. 1997
BCE1+	5^′^-AGTAGTTTTGTGTATAAACCA-3^′^	154	Adzhar et al. 1997

## Results

In the current research, eleven broiler flocks with age 5–7 weeks were selected for tracheal swab samples preparation. All broiler flocks were vaccinated against infectious bronchitis virus during the first week of life. Amplification of an expected DNA band (466 bp) from positive control as well as IBV positive swab samples indicated that the RT-PCR reaction has been performed correctly (Figure 1a). The results showed that 4 out of 11 (36.36%) of the sampled flocks were positive to IBV by RT-PCR. Specific nested PCR were performed on RT-PCR positive flocks and the Massachusetts was specific serotype of infectious bronchitis virus in broiler flocks of Zabol (Figure 1b).

## Discussion

Diagnosis of infectious bronchitis (IB) is commonly based on virus isolation in embryonated eggs, followed by immunological identification of the isolates. This procedure is time consuming and requires the use of specific polyclonal or monoclonal antibodies. Moreover, some isolates could be mixtures of different types of IBV that can confuse the interpretation of serotyping results. Reverse transcription PCR has been described previously using IBV, RNA extracted from allantoic fluid, and tracheal swabs. These techniques had been shown to be very efficient for the detection of IBV and for the identification of IBV types (Cavanagh et al. 1999; Handberg et al. 1999). It has been demonstrated that PCR and restriction analysis results are in agreement with the virus neutralization test (Kwon et al. 1993).

Molecular studies of IBV have shown that genetic and antigenic variants of this virus can emerge, mainly as a result of a few alterations or mutations in nucleotide sequences of the *S1* gene, while the majority of the IBV genome remains unaltered. The genetic variability in the *S1* subunit of the envelope spike glycoprotein gene represents an adaptive mechanism of the virus to immune selective pressures associated with intensive IBV vaccination and other management practices (Gelb et al. 1991; 1997). As a consequence, several serotypes of the virus are recognized and additional variant serotypes continue to evolve and cause disease episodes. Moreover, the vaccination of many broiler and layer flocks has been routinely made with a live attenuated vaccine against IB containing the strain H120. This strain belongs to the Massachusetts serotype and represents an additional problem for the discrimination of virulent field strains from this serotype, using RT-PCR or even RFLP techniques. Cavanagh et al. (1999) reported that when Massachusetts-type IB vaccines were applied at 1 day old in the hatchery, vaccine virus could later be detected in all broiler flocks tested by RT-PCR on swabs, with maximal amounts during the first week of life (Cavanagh et al. 1999).

Hadipour et al. (2011) used hemagglutination inhibition (HI) and ELISA to determine the rate of IBV antibodies titers in broiler flocks with respiratory symptoms in Iran, and the seroprevalence were reported 82.43%. Mahzounieh et al. (2006) have reported that 85.3% of the domestic village chickens in Iran had high titers of anti-IBV antibody without any clinical signs. In our study, 36.36% of the sampled broiler flocks were detected positive to IBV by RT-PCR and the Massachusetts was the identified serotype of the virus. However, infectious bronchitis is one of the main viral respiratory diseases of broilers in Zabol, southeast of Iran. Nouri et al. (2003) investigated the IBV serotypes in Fars Province of Iran and reported the 4/91 and Massachusetts serotypes. Seyfi-Abad Shapouri et al. (2002) have been reported the 4/91 serotype. Moreover, Shoushtari et al. (2008) revealed that the predominant circulating type of avian infectious bronchitis viruses in Iran during 1999–2004 is 793/B type IBV.

In conclusion, the present study confirms the existence of IBV by RT-PCR and the Nested PCR of positive samples demonstrated the Massachusetts strain. This is the first molecular evidence for the presence of infectious bronchitis virus and Massachusetts serotype in Zabol, southeast of Iran. Prevention of IB in chicken is based mainly on vaccination against the virus. In Iran, strain H120 of IBV has been used for a long time in broiler chicken flocks to achieve this goal. A vaccination program against IB fails when new strains of IBV emerge in a geographical region. Therefore, routinely monitoring of the existing IBV strains in a geographical region has been suggested to choose a suitable virus strain for vaccination.

## Authors’ original submitted files for images

Below are the links to the authors’ original submitted files for images. Authors’ original file for figure 1
