# Supplementary figures and images for: Detection of infectious bronchitis virus serotypes by reverse transcription polymerase chain reaction in broiler chickens
Source: Springerplus. 2013 Jan 31;2(1):36. doi: 10.1186/2193-1801-2-36 (PMC3579474; doi:10.1186/2193-1801-2-36)

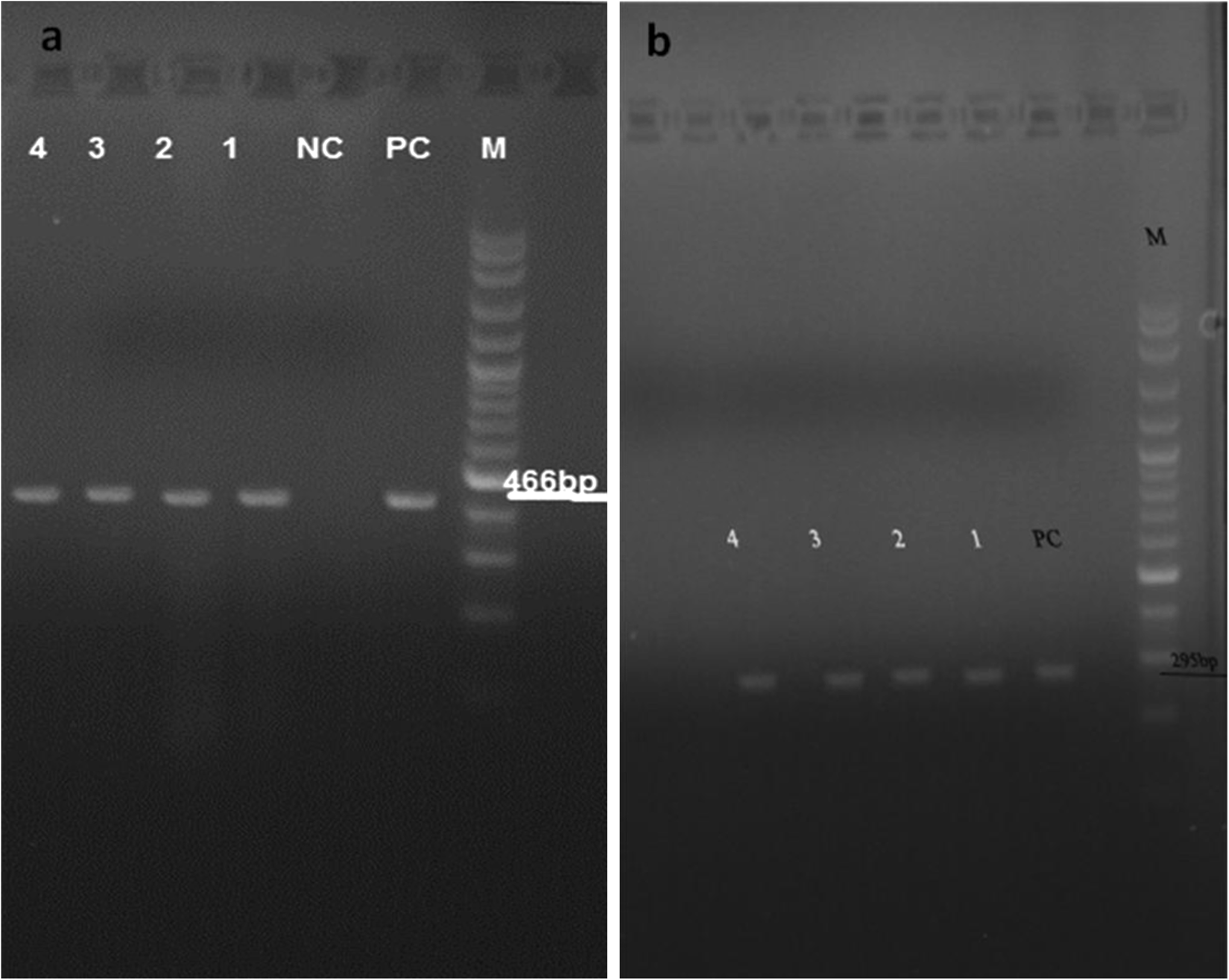

Supplement: Supplementary file 1 — Authors’ original file for figure 1 [file 40064_2012_83_MOESM1_ESM.tiff]
